# Characterization and Stabilization of a New ^64^Cu-Labeled Anti-EGFR Antibody NCAB001 for the Early Detection of Pancreatic Cancer with Positron Emission Tomography

**DOI:** 10.3390/pharmaceutics14010067

**Published:** 2021-12-28

**Authors:** Hiroki Matsumoto, Chika Igarashi, Tomoko Tachibana, Fukiko Hihara, Atsuo Waki, Ming-Rong Zhang, Sei Yoshida, Kenichiro Naito, Hiroaki Kurihara, Makoto Ueno, Kimiteru Ito, Tatsuya Higashi, Yukie Yoshii

**Affiliations:** 1 Institute for Quantum Medical Science, National Institutes for Quantum Science and Technology, Chiba 263-8555, Japan; matsumoto.hiroki2@qst.go.jp (H.M.); igarashi.chika@qst.go.jp (C.I.); tachibana.tomoko@qst.go.jp (T.T.); hihara.fukiko@qst.go.jp (F.H.); waki.atsuo@qst.go.jp (A.W.); zhang.ming-rong@qst.go.jp (M.-R.Z.); higashi.tatsuya@qst.go.jp (T.H.); 2 Department of Diagnostic Radiology, Kanagawa Cancer Center, Yokohama 241-8515, Japan; h-kurihara@kcch.jp; 3 Department of Research, NanoCarrier Co., Ltd., Tokyo 104-0031, Japan; yoshida@nanocarrier.co.jp (S.Y.); naito@nanocarrier.co.jp (K.N.); 4 Department of Gastroenterology, Kanagawa Cancer Center, Yokohama 241-8515, Japan; uenom@kcch.jp; 5 Department of Diagnostic Radiology, National Cancer Center Hospital, Tokyo 104-0045, Japan; kimito@ncc.go.jp

**Keywords:** ^64^Cu-NCAB001, ipPET, process development, stability, radiometal-antibody complex

## Abstract

Early diagnosis of pancreatic cancer using current imaging modalities remains challenging. We have developed a new approach to identify tumor lesions ≥ 3 mm in the pancreas by positron emission tomography (PET) with a new intraperitoneally administered ^64^Cu-labeled anti-epidermal growth factor receptor (EGFR) antibody (encoded as NCAB001), called ^64^Cu-NCAB001 ipPET. Generally, in clinical research, a radiometal-antibody complex must be prepared immediately before use at the imaging site. To make ^64^Cu-NCAB001 ipPET available to daily clinical practices in a sustainable way, the NCAB001-chelator conjugate and ^64^Cu-NCAB001 must be characterized and stabilized. NCAB001 was manufactured under cGMP conditions. NCAB001 was conjugated with a bifunctional chelator (p-SCN-Bn-PCTA), and the antibody-chelator conjugate (PCTA-NCAB001) was characterized by LC/MS and ELISA. Thereafter, to effectively manufacture ^64^Cu-NCAB001, we developed a new formulation to stabilize PCTA-NCAB001 and ^64^Cu-NCAB001. An average of three PCTA chelators were conjugated per molecule of NCAB001. The relative binding potency of PCTA-NCAB001 was comparable to cetuximab. The formulation consisting of acetate buffer, glycine, and polysorbate-80 stabilized PCTA-NCAB001 for a year-long storage. Additionally, this formulation enabled the stabilization of ^64^Cu-NCAB001 for up to 24 h after radiolabeling with a sufficient radioactivity concentration for clinical use. These results may accelerate the future use of ^64^Cu-NCAB001 ipPET in clinical settings for the early diagnosis and treatment of pancreatic cancer.

## 1. Introduction

The overall 5-year survival rate of pancreatic cancer patients is below 10%; early diagnosis and treatment of pancreatic cancer remains a significant clinical issue [1,2,3,4]. If tumor lesions could be detected at a resectable size (<1 cm), patient survival could be significantly improved by surgery; however, identification of small resectable pancreatic cancer remains challenging using current imaging modalities [5,6,7]. The epidermal growth factor receptor (EGFR) plays a key role in regulating cell proliferation, and its overexpression has been observed in several types of cancer, and especially in up to 90% of pancreatic cancer cases [8,9]. Therefore, EGFR is a good target for the imaging diagnosis of early pancreatic cancer with positron emission tomography (PET). Among the radionuclides applicable to PET imaging, we chose copper-64 (^64^Cu) to label EGFR since its half-life is suitable for the imaging of radiolabeled antibodies in humans. In addition, it can be easily produced using a biomedical cyclotron [10]. In our previous study, we labeled the clinically available anti-EGFR antibody cetuximab with ^64^Cu and intraperitoneally (ip) administered it to an orthotopic xenograft mouse model of small resectable (<1 cm) pancreatic cancer. PET imaging with ip-administered ^64^Cu-labeled cetuximab (^64^Cu-cetuximab ipPET) in mice enabled clear identification of tumor lesions ≥ 3 mm in the pancreas [11,12]. In contrast, we could not detect lesions ≥ 3 mm in diameter after intravenous (iv) administration of ^64^Cu-cetuximab or iv/ip administration of ^18^F-fluorodeoxyglucose (^18^F-FDG). To accelerate the development of this approach into clinical practice, we have established the efficient and sustainable manufacturing of a new anti-EGFR antibody encoded as NCAB001 (with the same amino acid sequence as cetuximab but different post-translational modifications such as the N-glycan profile) [13]. We used Chinese hamster ovary (CHO) cells instead of murine hybridoma cells (Sp2/O-Ag14), which has been used for the manufacturing of cetuximab [14], as the yield in CHO cells is higher than that in Sp2/O cells [15]. We have labeled NCAB001 with ^64^Cu and demonstrated that ^64^Cu-NCAB001 is protein-chemically, radiochemically, and biochemically comparable to ^64^Cu-cetuximab [13]. In addition, the ip-administration of ^64^Cu-NCAB001 to monkeys was safely conducted using ultrasound imaging [13]. On the basis of these promising findings, we have initiated the preclinical and pharmaceutical development of ^64^Cu-NCAB001 for the early diagnosis of pancreatic cancer with PET.

In this study, we characterized the NCAB001-chelator conjugate, as a final intermediate of radiopharmaceutical, by liquid chromatography-mass spectrometry (LC/MS) and the enzyme-linked immunosorbent assay (ELISA). Thereafter, we developed a new and efficient formulation for concurrently stabilizing antibody-chelator conjugate and ^64^Cu-labeled antibody. Generally, in clinical research, PET or single photon emission tomography (SPECT) with radiolabeled antibodies has been performed using radiotracers that are prepared at the imaging site immediately before use. For example, the anti-CD20 antibody ibritumomab labeled with ^111^In and ^90^Y, used in clinical practice [16], and ^64^Cu-cetuximab, used in preclinical evaluations [10,11,17,18,19], must be prepared on demand. There are two major limitations to the on-site preparation of radiolabeled antibodies in clinical settings. First, the conjugation of the bifunctional chelate for the radiometal and antibodies must be performed immediately before radiolabeling since the stability of antibody-chelator conjugates has not been systematically investigated. Second, radiolabeling has to be performed immediately before administration to patients since the radiochemical stability of the radiometal-antibody complexes has not been thoroughly evaluated. On-site preparation of the antibody-chelator conjugates followed by radiolabeling leads to (1) ineffective consumption of the antibody and bifunctional chelates; (2) limited availability of the radiometal-antibody complexes as final products, that is, delivery to distant imaging sites is not feasible; (3) difficulty in complying with the current quality standards for radiopharmaceuticals, and (4) excessive radiation exposure to the manufacturing personnel at the imaging site. These limitations have obstructed the potential use of radiolabeled antibodies as imaging agents for daily clinical practice.

There are two aspects to address these challenges, i.e., (1) a year-long storage of the antibody-chelator conjugates, and (2) stability of radiolabeled antibodies for 24 h delivery. The stability of the antibody-chelator conjugates mainly depends on the aggregation of proteins in aqueous solutions [20,21,22]. The stability of the radiolabeled antibodies is affected by reactive oxygen radicals formed by the radiolysis of water. Previous studies indicated that some pharmaceutical excipients exerted radical scavenging effects [23,24,25]. Many past efforts were made on these two challenges in separate manners. In contrast, we hypothesized that a single and suitable combination of the buffer and excipients could address these challenges concurrently. As a first step, for stabilizing the antibody-chelator conjugate for a year-long storage, several stock solutions were compared; acetate buffer with certain pharmaceutical excipients met the stabilization criteria. Second, we successfully showed that this stock solution enabled the stabilization of ^64^Cu-NCAB001 for up to 24 h after radiolabeling with a sufficient radioactivity concentration for clinical use. The formulation developed in this study may facilitate the future use of this promising diagnostic strategy for better outcomes related to pancreatic cancer.

## 2. Materials and Methods

### 2.1. Synthesis and Characterization of the NCAB001-PCTA Conjugate

The anti-EGFR antibody NCAB001 was manufactured under current good manufacturing practice (cGMP) conditions at Mycenax Biotech Inc. (Jhunan, Taiwan) [13]. For antibody conjugation, a NCAB001 solution (2 mg/mL in 50 mM borate buffer, pH 8.5) was prepared by buffer exchange with a Vivaspin ultrafiltration device (Sartorius, Göttingen, Germany). p-SCN-Bn-PCTA (Macrocyclics, Plano, TX, USA) was dissolved in dimethyl sulfoxide and added to the NCAB001 solution at a chelator-to-antibody molar ratio of 5:1, and the mixtures were incubated overnight at 37 °C. After conjugation, buffer exchanges were performed for PCTA-NCAB001 by ultrafiltration with three stock solutions, as described in Table 1, and the concentration of PCTA-NCAB001 was adjusted to 2 mg/mL.

The number of PCTA molecules conjugated to the antibody was determined using a LC/MS system. Briefly, a LC/MS system, which consisted of an LC (LC-30A, Shimadzu Corporation, Kyoto, Japan) and a mass spectrometer (maXis impact, Brucker Daltonics, Billerica, MA, USA), was used. Chromatographic separation was conducted using a MAbPac RP column (4 μm, 3.0 mm I.D. × 100 mm; ThermoFisher Scientific, Waltham, MA, USA), eluting with an acetonitrile-water gradient of 0.1% (*v*/*v*) formic acid. The elution of PCTA-NCAB001 was monitored using positive electrospray ionization. The mass spectra were deconvoluted by the maximum entropy method, and the molecular weight of PCTA-NCAB001 was determined.

The relative binding potency of NCAB001 and PCTA-NCAB001 against recombinant human EGFR were determined by ELISA using cetuximab (Merck Biopharma, Tokyo, Japan) as a reference standard. The wells in 96-well microplate were coated with recombinant human EGFR (R&D Systems, McKinley Place, NE, USA). After blocking with bovine serum albumin and washing, samples containing cetuximab, NCAB001, and PCTA-NCAB001 were added in triplicate and incubated at room temperature for 1 h. After the unbound antibodies had been removed, a peroxidase-conjugated anti-human IgG (Jackson ImmunoResearch Laboratories, West Grove, PA, USA) was added and incubated at room temperature for 1 h. The wells were washed to remove unbound reactants, tetramethylbenzidine (Biolegend, San Diego, CA, USA) was added, and the mixture left standing at room temperature for 15 min. Sulfonic acid was added to stop the reaction, and the absorption determined at 450 nm using a microplate reader (Spectra MAX M5, Molecular Devices, San Jose, CA, USA). 

### 2.2. Synthesis of ^64^Cu-NCAB001

^64^Cu was produced using a cyclotron and purified according to previously reported procedures [26]. Antibody conjugation and ^64^Cu labeling were conducted using previously described methods, with some modifications [27,28,29]. In short, for the antibody labeling with ^64^Cu, ^64^CuCl_2_ dissolved in acetate buffer (0.1 M, pH 6.0) was added to the three PCTA-NCAB001 stock solutions at a 3:1 ratio (vol:vol) and incubated for 1 h at 40 °C. The radiochemical purity of ^64^Cu-NCAB001 was determined using radio-thin layer chromatography (radio-TLC). To separate the free [^64^Cu] copper ion from ^64^Cu-NCAB001, a silica gel 60 plate (Merck Millipore, Burlington, MA, USA) and a mobile phase of methanol:water (80:20, *v*/*v*) were used. The retention factor of ^64^Cu-NCAB001 on TLC were 0–0.1, which is identical to that of the reference standard cetuximab [13]. The retention factor of the radio-chemical impurities of ^64^Cu-NCAB001 on TLC were identical to that of the free [^64^Cu] copper ion (0.8). The radioactivity on TLC plates was analyzed using a bioimaging analyzer (FLA-7000, GE Healthcare, Marlborough, MA, USA), and the relative activity ratios were calculated as the fraction with intact ^64^Cu-NCAB001 relative to the total radioactivity. A radiochemical purity ≥ 95% was set as a target specification criterion for the ^64^Cu-NCAB001 product.

### 2.3. Stability of PCTA-NCAB001 Conjugate in the Stock Solutions

PCTA-NCAB001 dissolved in the three stock solutions (Table 1) was stored at 4 °C for 12 months and labeled with ^64^Cu at the time of preparation (0 months) and at 3, 6, 8, 9, and 12 months thereafter. ^64^Cu (37 MBq) was labeled with 20 μg of PCTA-NCAB001 in a total volume of 40 μL. The radiochemical purities at the time of radiolabeling were determined using radio-TLC and compared among the stock solutions during the storage period; the effect of the stock solutions on the stability of PCTA-NCABB001 was evaluated.

The cell-binding properties of ^64^Cu-NCAB001 prepared with each stock solution were also compared. Human colon cancer HCT116 cells (CCL-247; American Type Cell Collection) were selected to evaluate the binding affinity of ^64^Cu-NCAB001 to EGFR since this cell line expresses EGFR sufficiently for this purpose [10]. HCT116 cells were cultured as reported previously [10]. HCT116 cells (6.25 × 10^5^ cells) were diluted in PBS with 1% bovine serum albumin (BSA) (Sigma-Aldrich) and incubated with ^64^Cu-NCAB001 in triplicate on ice for 1 h. After washing, the radioactivity bound to the cells was measured using a γ-counter (1480 Automatic gamma counter Wizard 3; PerkinElmer, Waltham, MA, USA) and compared among the ^64^Cu-NCAB001 conjugates prepared from each stock solution. Cetuximab was used as the reference standard.

### 2.4. Effect of the Stock Solution on the Stability of ^64^Cu-NCAB001 after Radiolabeling

PCTA-NCAB001 was dissolved in the three stock solutions (Table 1) and stored at 4 °C for 3 and 12 months. PCTA-NCAB001 (20 μg) in each stock solution was labeled with ^64^Cu (37 MBq) in a final product volume of 40 μL. The radiochemical purities were determined using radio-TLC at the time of radiolabeling (0 h) and at 0.5, 1, 3, and 24 h thereafter. The results were compared, and the effect of the stock solutions on the stability of ^64^Cu-NCAB001 after radiolabeling was evaluated.

### 2.5. Radiolabeling of ^64^Cu-NCAB001 for Clinical Use

Among the three stock solutions (Table 1), Solution C was chosen for further evaluation because it showed the most promising effect on the stabilization of ^64^Cu-NCAB001 with respect to radiochemical, chemical, and biochemical stability. The PCTA-NCAB001 dissolved in Solution C was stored at 4 °C for 12 months and radiolabeled to obtain a sufficient radioactivity concentration for clinical use. We labeled 130, 370, and 520 MBq of ^64^Cu with 90 μg of PCTA-NCAB001 in a total volume of 5 mL. The radiochemical purities of these preparations were confirmed to be > 95% by radio-TLC at the time of radiolabeling. Cell-binding assays were performed in triplicate, and the biochemical stability of ^64^Cu-NCAB001 was evaluated. We used ^64^Cu-NCAB001 (37 MBq ^64^Cu with 20 μg PCTA-NCAB001 in a total volume of 40 μL) as a reference.

### 2.6. Statistical Analysis

ELISA and cell-binding assay data were expressed as the mean with corresponding standard deviations. *p* values were calculated using analysis of variance for comparison of multiple groups. In the case of heterogenous group variances, the Steel–Dwass test was performed. *p* values less than 0.05 were considered to indicate statistical significance.

## 3. Results and Discussion

### 3.1. Characterization of PCTA-NCAB001 Conjugate

**Figure 1:**
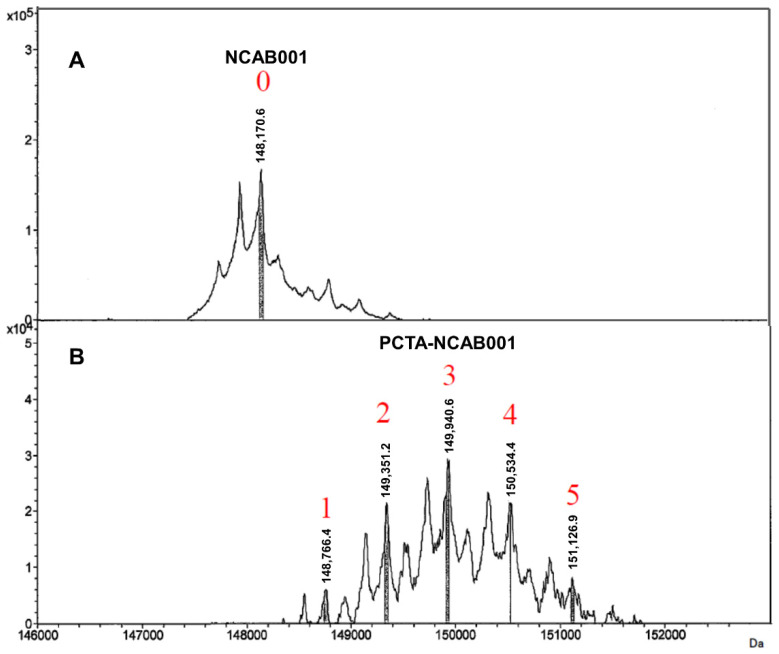
Deconvoluted mass spectra of NCAB001 (**A**) and PCTA-NCAB001 (**B**). The numbers on these spectra denote the numbers of PCTA molecules conjugated to NCAB001 (average of 3, range from 1 to 5).

**Figure 2:**
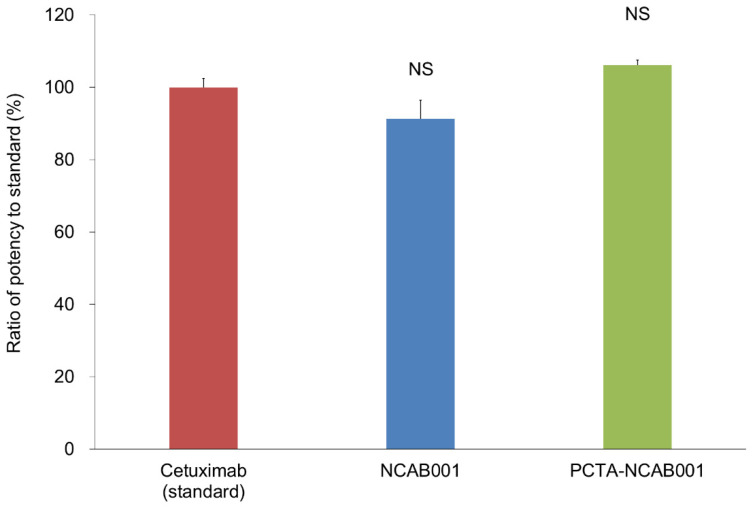
The relative binding potency of NCAB001 and PCTA-NCAB001 against recombinant human EGFR was determined by the enzyme-linked immunosorbent assay (ELISA). Cetuximab was used as the reference standard. NS = not significant vs. standard.

The anti-EGFR antibody NCAB001 was manufactured under cGMP conditions [13]. NCAB001 and 3,6,9,15-tetraazabicyclo[9.3.1]pentadeca-1(15),11,13-triene-4-*S*-(4-isothiocyanatobenzyl)-3,6,9-triacetic acid (p-SCN-Bn-PCTA) were conjugated, and the number of PCTA molecules conjugated to NCAB001 was determined by LC/MS. An average of three PCTA molecules were conjugated to NCAB001 (in the range of 1 to 5; Figure 1). The relative binding potency of NCAB001 and the antibody-chelator conjugate, PCTA-NCAB001, against recombinant human EGFR was determined by ELISA. NCAB001 and PCTA-NCAB001 had a comparative potency to cetuximab, which was used as the reference standard (Figure 2). These results support that PCTA-NCAB001 can be used as the final intermediate for ^64^Cu labeling; therefore, we determined suitable stock solutions for the long-term storage of PCTA-NCAB001.

### 3.2. Selection of the Stock Solution of PCTA-NCAB001 for Year-Long Storage

**Figure 3:**
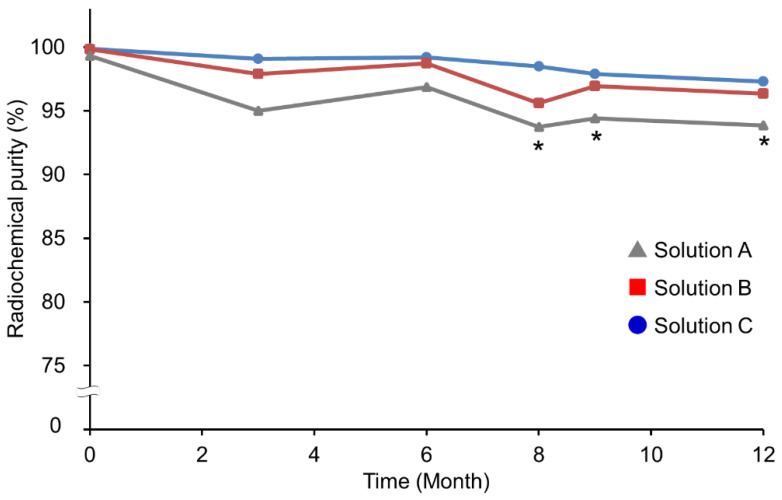
Effect of the stock solutions on the stability of PCTA-NCAB001 conjugates. We prepared radiolabeled ^64^Cu-NCAB001 using PCTA-NCAB001 stored at 4 °C for 12 months in Solutions A, B, and C (compositions in Table 1) and compared the radiochemical purity of each condition as the stability index of PCTA-NCAB001; * denotes radiochemical purity ˂ 95%.

We attempted to stabilize the PCTA-NCAB001 for a year-long storage. The stabilizing effect of acetate buffer and some pharmaceutical excipients was investigated, using physiological saline as a control (Solutions A–C; Table 1). Figure 3 presents the radiochemical purities of ^64^Cu-NCAB001 prepared from PCTA-NCAB001 stored at 4 °C for 12 months in the three stock solutions. Radiochemical purity was determined using radio-TLC at the time of radiolabeling. At the time of preparation (0 months), with all three solutions, ^64^Cu-NCAB001 prepared from PCTA-NCAB001 exhibited more than 95% radiochemical purity, with a specific activity of 1.1 to 1.7 GBq/mg, as reported previously [13]. After eight months of storage, ^64^Cu-NCAB001 prepared from PCTA-NCAB001 stored in Solution A (saline) showed a decline in radiochemical purity to ˂ 95%. In contrast, ^64^Cu-NCAB001 prepared from PCTA-NCAB001 stored in Solutions B and C maintained its property, achieving a radiochemical purity of ≥ 95%. In particular, Solution C resulted in a more stable effect than Solution B.

**Figure 4:**
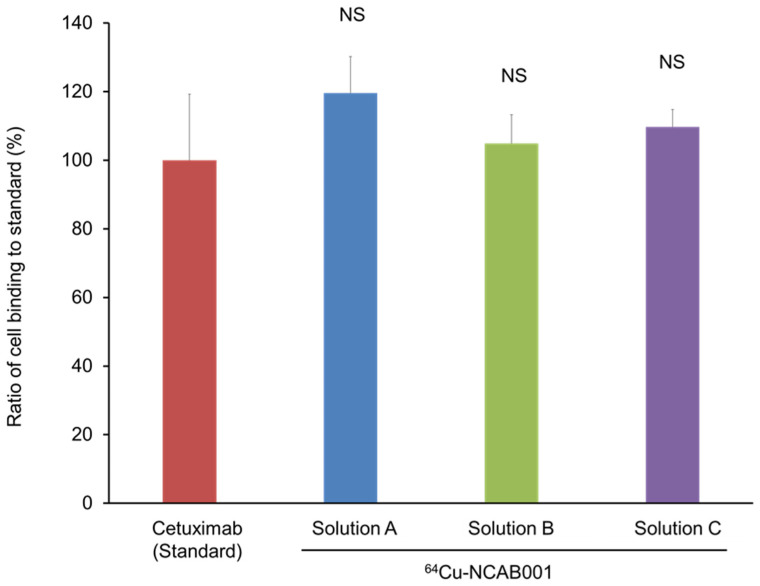
Effect of the stock solutions on the cell-binding properties of ^64^Cu-NCAB001. PCTA-NCAB001 that was dissolved in Solutions A, B, and C (compositions in Table 1) and stored at 4 °C for 12 months was labeled with ^64^Cu. The binding properties to HCT116 cells were evaluated using cetuximab as the reference standard. NS = Not significant vs. standard.

Cell binding assays of ^64^Cu-NCAB001 prepared with PCTA-NCAB001 dissolved in the three stock solutions (Table 1) and stored at 4 °C for 12 months were conducted using cetuximab as the reference standard (Figure 4). All ^64^Cu-NCAB001 preparations based on PCTA-NCAB001 stored in the three solutions at 4 °C for 12 months exhibited similar cell-binding properties compared to freshly prepared ^64^Cu-cetuximab.

These results suggest that PCTA-NCAB001 stored in Solution C is radiochemically and biochemically stable for a year-long period. A previous study has shown that acetate buffer has a lower aggregation propensity than phosphate and citrate buffers; this aggregation dependency is based on the specific molecular interaction between the buffer and IgG, rather than the ionic strength [20]. Polysorbate-80 is used in the formulation of biotherapeutic products as a nonionic surfactant to influence the stability of biopharmaceutical products [21]. Additionally, amino acids are commonly used to prevent protein aggregation by influencing the protein folding pathway [22]. We selected glycine as a pharmaceutical excipient for our formulation because it exhibits favorable properties such as water solubility and neutral acidity or alkalinity in physiological aqueous solutions. We hypothesized that the combination of acetate buffer with polysorbate-80 and glycine would have a beneficial effect in stabilizing the antibody-chelator conjugate compared with the saline. As shown in Figure 3, acetate buffer (Solution B in Table 1) showed a protective effect against the decline in radiochemical purity during the year-long storage which was seen in saline (Solution A in Table 1). Moreover, addition of polysorbate-80 and glycine to acetate buffer (Solution C in Table 1) further protected against the decline in radiochemical purity. This suggests that the aggregation of PCTA-NCAB001 progressed gradually in the saline and that acetate buffer, polysorbate-80, and glycine contributed to maintaining the radiolabeling yield for a year by acting as stabilizers against the aggregation of PCTA-NCAB001.

### 3.3. Effect of the Stock Solution on the Stability of ^64^Cu-NCAB001 after Radiolabeling

**Figure 5:**
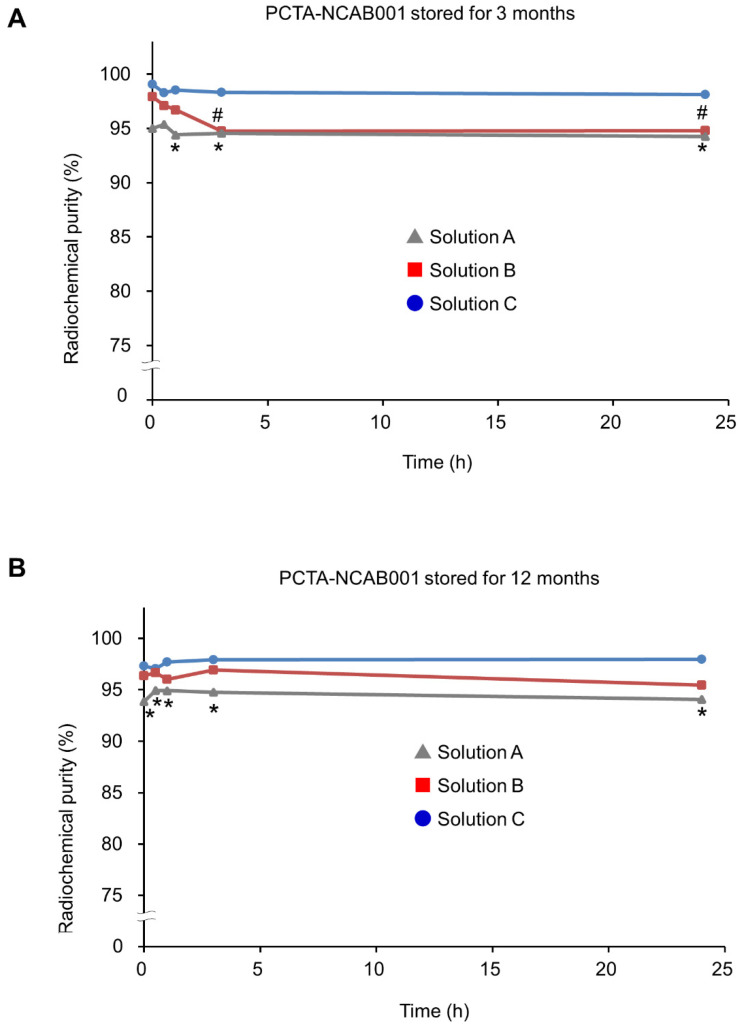
Effect of the stock solutions on the stability of ^64^Cu-NCAB001. We prepared radiolabeled ^64^Cu-NCAB001 using PCTA-NCAB001 stored at 4 °C for 3 months (**A**) and 12 months (**B**) in Solutions A, B, and C (composition in Table 1) and compared the radiochemical purity of each preparation; * and # denote a radiochemical purity less than 95%.

We then attempted to stabilize ^64^Cu-NCAB001 for up to 24 h after radiolabeling. PCTA-NCAB001 was dissolved in the three stock solutions (Table 1), stored at 4 °C for 3 and 12 months, and labeled with ^64^Cu; the radiochemical purities were determined using radio-TLC (Figure 5). After three months of storage (Figure 5A), PCTA-NCAB001 in Solution A (saline) exhibited a radiochemical purity of ˂ 95% at 1 h after radiolabeling and thereafter. PCTA-NCAB001 in Solution B presented a radiochemical purity of ≥ 95% up to 1 h after radiolabeling, which declined to ˂ 95% at 3 and 24 h. In contrast, Solution C achieved a radiochemical purity of ≥ 95% up to 24 h after radiolabeling. Similar results were obtained using PCTA-NCAB001 stored for 12 months (Figure 5B).

A previous study indicated that hydroxyl radicals, formed during the radiolysis of water, react with the nitrogen atom of glycine [23], suggesting the radical scavenging activity of glycine. Another study showed that polysorbate-80 exerts a radical scavenging effect [24]. We have reported that amino acids exert a protective effect against the radiolysis of ^64^Cu-ATSM [25]. The results in this study suggest that the protective effect of acetate buffer is sufficient for PCTA-NCAB001 (Figure 3) but not sufficient against radiolysis of ^64^Cu-NCAB001 (Figure 5). In contrast, acetate buffer with glycine and polysorbate-80 achieved a radiochemical purity of ≥ 95% up to 24 h after radiolabeling (Figure 5). Our present study, together with these previous reports [23,24,25], suggests that glycine and polysorbate-80 exhibit protective effects against radiolysis of ^64^Cu-NCAB001.

### 3.4. Radiolabeling of ^64^Cu-NCAB001 for Clinical Use

**Figure 6:**
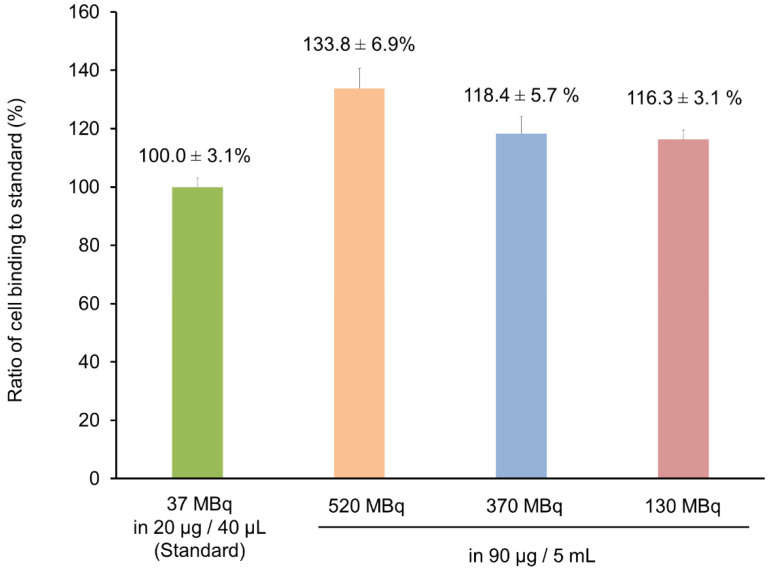
Cell binding of ^64^Cu-NCAB001 prepared at clinically feasible radioactivity concentrations. PCTA-NCAB001 dissolved in Solution C (composition in Table 1) and stored at 4 °C for 12 months was radiolabeled with ^64^Cu, and cell-binding assays were performed. Cell binding of ^64^Cu-NCAB001 at clinically feasible radioactivity concentrations up to 520 MBq for 90 μg of PCTA-NCAB001 in 5 mL was evaluated using the experimental preparation with 37 MBq for 20 μg of PCTA-NCAB001 in 40 μL as a reference standard.

Lastly, the stock solutions were evaluated for use in radiolabeling, for achieving radioactivity concentrations sufficient for clinical use. PCTA-NCAB001 was dissolved in Solution C, stored at 4 °C for 12 months, and radiolabeled with ^64^Cu, followed by cell-binding assays (Figure 6). The clinically feasible radioactivity concentrations of up to 520 MBq for 90 μg of PCTA-NCAB001 in 5 mL at the time of radiolabeling did not alter the cell-binding property of ^64^Cu-NCAB001, relative to the cell-binding property of the experimental preparation (37 MBq for 20 μg of PCTA-NCAB001 in 40 μL).

A previous study reported that 130 MBq of ^64^Cu-DOTA-trastuzumab was feasible for the identification of HER2-positive lesions by PET in patients with primary and metastatic breast cancer [30]. Although further careful preclinical studies are required, 130 MBq of ^64^Cu-NCAB001 would be a feasible starting point for the first-in-patient clinical study of patients with early-stage pancreatic cancer. The results in this study can be applied in the manufacturing of ^64^Cu-NCAB001 as radiopharmaceutical for clinical trials towards the early detection of pancreatic cancer using clinical PET imaging on a daily basis.

There is a limitation in this study. We evaluated the stability of PCTA-NCAB001 by means of radiochemical and biochemical measurements. At the process development stage of ^64^Cu-NCAB001, these criteria were sufficient to move to the next step. To bring this radiotracer to the clinical development stage, protein chemical evaluations, involving mass spectrometry, electrophoresis, and size exclusion chromatography, are in progress to establish a quality control program for PCTA-NCAB001.

## 4. Conclusions

We characterized the conjugate of a new anti-EGFR antibody NCAB001 with p-SCN-Bn-PCTA as the final intermediate of the ^64^Cu-NCAB001 radiopharmaceutical. We developed the new formulation to enable the long-term storage of PCTA-NCAB001. Additionally, this formulation is useful for the stabilization of ^64^Cu-NCAB001 for 24 h after radiolabeling. These results may accelerate the future use of ^64^Cu-NCAB001 ipPET in clinical applications for the early diagnosis and treatment of pancreatic cancer. Chemical and preclinical development programs are in progress.

## Figures and Tables

**Table 1:** Formulation of the stock solutions.

Stock Solutions	Ingredients
Solution A	Physiological saline
Solution B	0.1 M acetate buffer (pH 6.0)
Solution C	0.1 M acetate buffer (pH 6.0) containing 100 mM glycine and 76.3 μM polysorbate-80

## Data Availability

Data are within the article.
